# Trigonella Foenum-Graecum Seeds Lowers Postprandial Blood Glucose in Overweight and Obese Individuals

**DOI:** 10.1155/2014/964873

**Published:** 2014-09-03

**Authors:** Sathyasurya Daniel Robert, Aziz Al-Safi Ismail, Wan Ishak Wan Rosli

**Affiliations:** ^1^Dietetics Program, School of Health Sciences, Health Campus, Universiti Sains Malaysia, Kubang Kerian, 16150 Kota Bharu, Kelantan, Malaysia; ^2^Department of Community Medicine, School of Medical Sciences, Health campus, Universiti Sains Malaysia, Kubang Kerian, 16150 Kota Bharu, Kelantan, Malaysia; ^3^Nutrition Program, School of Health Sciences, Health Campus, Universiti Sains Malaysia, Kubang Kerian, 16150 Kota Bharu, Kelantan, Malaysia

## Abstract

This study determined the effects of fenugreek on postprandial plasma glucose (PPG) and satiety among overweight and obese individuals. Fourteen subjects were studied in the morning after overnight fasts on four separate occasions. Glycaemic responses elicited by 50 g carbohydrate portions of white bread and jam with or without 5.5 g of fenugreek and fried rice with or without 5.5 g fenugreek were determined over 2 h. The primary endpoint was the incremental area under the plasma glucose response curve (IAUC). Adding fenugreek to both foods significantly reduced the IAUC compared to the food alone: white bread and jam, 180 ± 22 versus 271 ± 23 mmol × min/L (*P* = 0.001); fried rice, 176 ± 20 versus 249 ± 25 mmol × min/L (*P* = 0.001). Fenugreek also significantly reduced the area under the satiety curve for white bread with jam (134 ± 27 versus 232 ± 33 mm × hr, *P* = 0.01) and fried rice (280 ± 37 versus 379 ± 36 mm × hr, *P* = 0.01). It is concluded that fenugreek significantly decreased the PPG response and increased satiety among overweight and obese individuals.

## 1. Introduction

Excessive body weight increases the risk for many chronic diseases. In 2008, more than 1.4 billion adults worldwide were overweight and of these more than 500 million were obese [1]. By 2015 it is expected that approximately 2.3 billion adults around the world will be overweight and that at least 700 million will be obese [2]. The rising trend of obesity is evident in most countries including Malaysia, where the prevalence of overweight and obesity is about 29% and 14%, respectively [3]. This obesity epidemic is mainly due to excess energy intake and decreased energy expenditure [4–6]. Hence identification of natural functional foods that could increase the satiety and control the surge in the postprandial blood glucose (PPG) may promote more effective weight management and reduce the risk of overweight or obese individuals for developing chronic metabolic diseases [7, 8]. Trigonella foenum-graecum (fenugreek) has long been used as a cooking spice and as a traditional medicine for its therapeutic properties [9, 10]. Preliminary animal and human trials suggest possible hypoglycemic properties of fenugreek when taken orally [11]. Thus, fenugreek may be an essential ingredient in cooking as well as for the development of novel highly satiating food products with a low glycaemic index (GI). Recent research findings indicate that frequent consumption of foods with low GI reduces the risk of developing chronic diseases [12–15]. Rice and bread are commonly consumed by the Malaysian population [16]. Therefore, incorporating fenugreek in these foods may provide health benefits. However, no prior Malaysian studies are done to determine the effect of fenugreek on glycaemic control or satiety among the overweight or obese individuals. Hence the purpose of this study was to examine the effects of untreated fenugreek seed powder on postprandial glycaemic responses and satiety in overweight and obese Malaysian individuals.

## 2. Materials and Methods

### 2.1. Subjects

Sixteen overweight or obese subjects (7 men and 9 women) aged 32–52 years were recruited through the advertisements posted in the intranet web mail of the Universiti Sains Malaysia. Subjects were excluded based on the following criteria: BMI < 25 kg/m^2^, currently pregnant or lactating, intended or unintended weight loss of >5 kg in the prior 3 months, history of binge-eating disorder, smoking or alcohol abuse, diabetes, renal or hepatic diseases, use of weight loss, lipid-lowering, antihypertensive or anti-inflammatory steroid medications, and use of fiber supplements. Two subjects were eliminated during the study process because their postprandial glycaemic response was >16.9 mmol/L (304.2 mg/dL). Hence the study was completed with 14 subjects. The Universiti Sains Malaysia Ethical Committee approved the study. All subjects gave written, informed consent after reviewing the study procedures. The study was conducted at the Dietetics Laboratory, School of Health Sciences, Universiti Sains Malaysia.

### 2.2. Control and Test Foods

Fenugreek seeds (TESCO; Kota Bharu) were heated in a microwave oven for one minute and ground in a blender. The ground seeds were sieved to obtain a fine powder which was then stored in an air tight container. Fenugreek seed powder was added to a white bread and strawberry jam sandwich by mixing 5.5 g powder into 15.6 g jam which was spread onto 83.7 g white bread. Fenugreek seed powder was added to fried rice as follows: 62.3 g white rice was cooked in a rice cooker for 25–30 min and transferred to a hot frying pan containing 5 g of oil. Chicken flavoured seasoning (8.5 g) and 5.5 g fenugreek seed powder were added to the rice, stirred, and sautéed for 3 min. The nutrient composition of the test foods is shown in Table 1.

### 2.3. Feeding Protocol

Prior to the main study a pilot study was done with 3 subjects in order to determine the taste tolerance of fenugreek powder and its effect on the PPG response. No effect was noticed with 2 g of fenugreek seed powder per 50 g carbohydrate portion of white bread and jam. However, considerable reduction in the PPG was noted when the white bread and jam contained 5.5 g of fenugreek seed powder. In addition the subjects were able to tolerate the bitter taste of the fenugreek at this dose. Therefore, the main study had a crossover design in which 14 subjects participated on four separate occasions after a 10–12 hr overnight fast when they consumed, in random order, one of the following test meals: 50 g carbohydrate from white bread and jam alone or with 5.5 g fenugreek seed powder or 50 g carbohydrate from fried rice alone or with 5.5 g fenugreek seed powder. All subjects underwent 4 tests on separate mornings with at least 10 to 15 days between successive testing sessions. On each incident subjects gave a fasting blood sample and then consumed one of the 4 study meals within 10 to 13 minutes; further blood samples were collected at 15, 30, 45, 60, 90, and 120 minutes after starting to eat. Study meals were served with 250 mL of water. Capillary finger-prick blood samples (11 drops) were collected into 1.5 mL eppendorf tubes containing fluoride oxalate and centrifuged to obtain plasma, which was stored at –20°C prior to analysis. Plasma glucose was measured by using a Randox glucose GOD/PAP autoanalyzer which uses the glucose oxidase method. All subjects were asked to complete a satiety score sheet before consuming each test meal and after every blood sample thereafter for 2 h using a 6-point scale on which 0 was “extremely hungry” and 6 was “extremely full” [17]. Upon completion of the test meals, subjects rated the palatability on a Likert scale on which a score of 1 was “strongly dislike,” 2 was “dislike,” 3 was “neutral,” 4 was “like,” and 5 was “strongly like” [18].

### 2.4. Statistical Analysis

Data analysis was conducted using Microsoft Excel Spreadsheets and Graph Pad Prism version 5 (GraphPad Software, San Diego, CA, USA). The IAUC values and satiety scores were subjected to repeated measures ANOVA and, after demonstrating significant heterogeneity, the least significant differences between individual means were obtained by Bonferroni multiple comparisons. A value of *P* < 0.05 was considered significant. Results are expressed as means ± SEM.

## 3. Results

The 14 subjects (7 women and 7 men) who completed the study were aged 38.6 ± 1.4 years and had a BMI of 29.9 ± 1.3 kg/m^2^. Among the subjects 28.6% (3 men and 1 woman) were obese and the rest were overweight. Their mean systolic blood pressure was 122.6 ± 3.1 mm Hg and diastolic blood pressure was 78 ± 1.7 mm Hg.

### 3.1. Blood Glucose Responses

**Figure 1:**
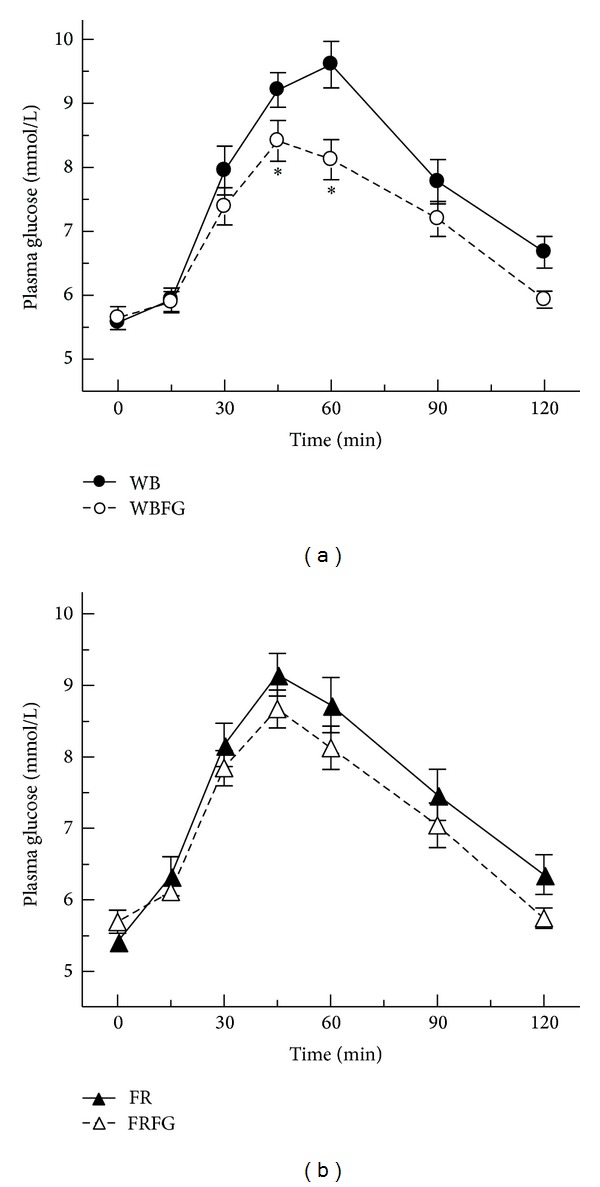
(a) Mean plasma glucose response of white bread and white bread with fenugreek. Values are means ± SEM (*n* = 14). Comparison of plasma glucose concentrations (*P < 0.05). WB: white bread; WBFR: white bread with fenugreek. (b) Mean plasma glucose response of fried rice and fried rice with fenugreek. Values are means ± SEM (*n* = 14). Comparison of plasma glucose concentrations. FR: fried rice; FRFG: fried rice with fenugreek.

Plasma glucose after consuming white bread with fenugreek was significantly lower at 45 min and 60 min when compared to white bread alone (Figure 1(a)). Plasma glucose after fried rice with fenugreek tended to be lower at 45 min and 60 min when compared to fried rice alone but the difference was not statistically significant (Figure 1(b)). However, significant differences were seen in IAUC between the control and fenugreek-enriched foods for both bread and rice (*P* = 0.001) (Table 2).

### 3.2. Satiety and Taste

The satiety incremental area under the curve (SIAUC) of both test foods was significantly higher than the SIAUC of the control foods (*P* = 0.01) (Table 2). Palatability ratings of white bread with fenugreek showed that 57.1% of the subjects choose the option dislike, 28.6% choose neutral, and 14.3% choose like. Whereas the palatability ratings of fried rice with fenugreek revealed that 14.3% of the subjects choose the option dislike, 21.4% choose neutral, and 64.3% choose like. Paired *t*-test showed that there were no statistical differences in palatability score between the 2 test foods, *P* = 1.0.

## 4. Discussion

This crossover study demonstrated that incorporation of natural fenugreek seed powder to the test foods lowered the surge in PPG response and increased satiety in overweight and obese subjects. It was noted that there was a reduction of 14.4 mg/dL (PPG) at 45 min and a reduction of 27 mg/dL (PPG) at 60 min after the consumption of white bread with fenugreek, when compared with that of the control food. On the other hand, it was noted that after the consumption of fried rice with fenugreek the PPG decreased by 10.8 mg/dL at 60 min, when compared to the control food, but it was not significantly lower. A previous study by Mathern et al. found no reductions in PPG in response to a beverage supplemented with 8 g of isolated fenugreek fiber among obese subjects [19]. In another study, Sharma and Raghuram found that 100 g of defatted fenugreek seed powder, when given for 20 days, produced a significant fall in fasting blood glucose levels and an improvement in glucose tolerance test among subjects with type 2 diabetes [20]. Results from several additional studies also suggest that fenugreek seeds may improve glycaemic control in patients with type 2 diabetes [21–24]. However, in all those studies the patients took their regular glucose lowering medications. The hypoglycemic action of fenugreek has been related to several processes. In vitro studies demonstrated that the amino acid 4-hydroxyisoleucine in fenugreek seeds increased insulin release from human and rat pancreas. This amino acid acted only on pancreatic beta cells, but the levels of other hormones such as somatostatin and glucagon were not changed [10]. Other human studies reported that fenugreek seeds increased the number of insulin receptors, thereby decreasing the postprandial area under the curve [10]. Fenugreek seeds can also exert hypoglycemic effects by inhibiting the activities of alpha-amylase and sucrase, the two intestinal enzymes that are involved in carbohydrate digestion and absorption [10]. In clinical studies the hypoglycemic action of fenugreek has been attributed to its content of galactomannan, a water soluble fiber, which inhibits glucose absorption and slows gastric emptying [25–29]. On the other hand the satiety area under the curve after the test foods was significantly higher than that after the control foods. The increase in satiety may be due to the presence of the soluble fiber galactomannan in fenugreek. Galactomannan has gel forming characteristics which reduces gastric emptying [25]. It has been reported that 8 g of fenugreek extract, providing 7.2 g total fiber (78.9% soluble fiber), increased satiety and reduced hunger among obese subjects [19]. However, in this study we noted that addition of fenugreek seed powder to the test foods increased its total dietary fiber content by 2.18 g, which increased satiety. Several studies have stated that protein can promote satiety [30–32]. Protein induced satiety may be due to increase in thermogenesis, increase in the concentrations of the appetite suppressing hormones such as glucagon like peptide-1, cholecystokinin, and peptide tyrosine tyrosine, and decrease in appetite stimulating hormones such as ghrelin [33–38]. In a single blinded crossover design study, it was noted that satiety was increased after the subjects consumed a higher protein lunch containing 25% of the energy as dietary protein [39]. But in this study the effect of protein on satiety may be minor, because the addition of fenugreek to the test foods increased their protein content by merely 13% (white bread) and 10.2% (rice) of the energy.

In the earlier studies, defatted or isolated or extracted fenugreek was used as experimental food [20]. In contrast, untreated fenugreek seed powder was given to the subjects of this study. Processed fenugreek seed powder or any processed functional food may not be easily available for the general public. But our study showed that untreated fenugreek (functional food) can easily be obtained from the food market and by following simple cooking methods can be added in the daily diet, which is the strength of this study. The subjects of this study were able to tolerate the taste of the fenugreek well when it was added with fried rice than when it was added with white bread and jam. This shows that the bitterness of the fenugreek can be masked when it is added to spicy foods. The limitation of this study is the bitterness of the fenugreek which may limit its daily use.

## 5. Conclusion

In conclusion, addition of 5.5 g of untreated fenugreek seed powder to rice or bread containing 50 g available carbohydrate reduced PPG responses and increased satiety in overweight and obese individuals. As obesity is a component of metabolic syndrome which eventually leads to type 2 diabetes, incorporation of fenugreek in the daily diet may assist with weight management and hence may help prevent or delay the onset of chronic diseases.

Further research can be done to measure insulin index in order to learn more about the mechanism of action. In addition novel food products enriched with fenugreek can be developed.

## Figures and Tables

**Table 1:** Composition of control and test foods.

Food	Portion size (g)	Protein (g)	Fat (g)	Dietary fiber (g)	Available carbohydrate^a^ (g)	Energy (K*·*cals)
White bread and jam^†^	99.3	7.2	2.2	1.3	50	257.3
White bread and jam with fenugreek^‡^	104.8	8.9	2.6	3.5	50	273.7
Fried rice^†^	62.3	5.2	5.0	3.6	50	254.9
Fried rice with fenugreek^‡^	67.8	6.9	5.4	5.8	50	271.4
Fenugreek (per 5.5 g)	—	1.7	0.4	2.2	0.4	16.4

^a^Available carbohydrates are calculated as follows: 100 − (moisture + protein + fat + dietary fiber + ash).

^†^Control foods.

^‡^Test foods.

**Table 2:** Incremental area under the curve and satiety area under the curve of test foods and control foods.

Foods	Area under the curve (mmol × min/L)	Satiety area under the curve (mm × hr)
White bread and jam^†^	271 ± 23	134 ± 27
White bread and jam with fenugreek^‡^	180 ± 22∗	232 ± 33∗
Fried rice^†^	249 ± 25	280 ± 37
Fried rice with fenugreek^‡^	176 ± 20∗	379 ± 36∗

Mean ± SEM.

∗Mean values within a column are significantly different from the respective control foods (*P* < 0.05).

^†^Control foods.

^‡^Test foods.
